# The Forest and the Trees: How Population-Level Health Protections Sometimes Fail the Individual

**DOI:** 10.1289/ehp.125-A65

**Published:** 2017-03-31

**Authors:** Nate Seltenrich

**Affiliations:** Nate Seltenrich covers science and the environment from Petaluma, CA. His work has appeared in *High Country News*, *Sierra*, *Yale Environment 360*, *Earth Island Journal*, and other regional and national publications.

Beijing residents wearing face masks to protect themselves against extreme air pollution.^1^ Residents of small towns such as Mooringsport, Louisiana,^2^ and Coal Mountain, Virginia,^3^ buying bottled water or filters to avoid unsafe water. For epidemiologist Barbara Hoffmann of Heinrich-Heine-University in Dusseldorf, Germany, such examples of individual efforts to evade environmental pollutants raise serious ethical questions. “Only some people can afford to take preventive measures,” she says. “Others don’t have the means to do it.”

**Figure:**
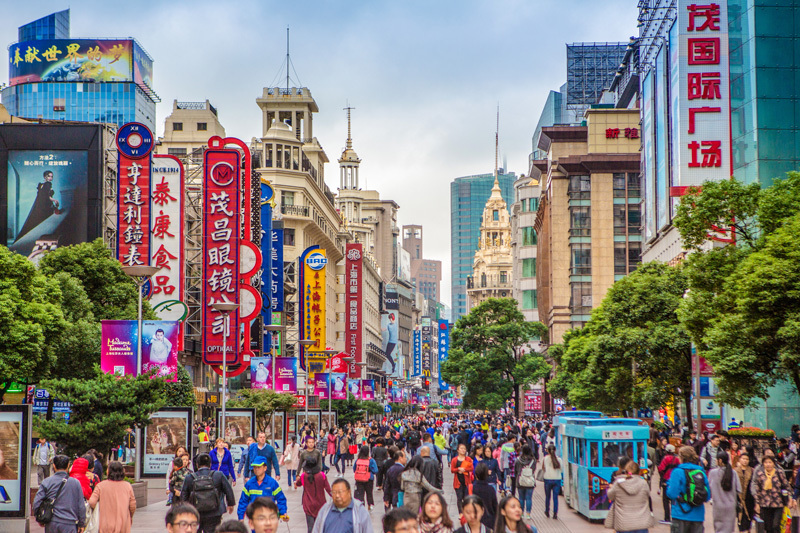
Burdening individuals with the responsibility of reducing their own environmental exposures is not only unreliable in terms of health protection but also a contributor to environmental injustice. © Raga/Getty Images

As a result, Hoffmann says, burdening individuals with the responsibility to reduce their own environmental exposures can create or worsen environmental injustice, in which harmful exposures are inequitably distributed across a population.

Moreover, Hoffmann fears that the tacit acceptance by a population of individualized efforts may limit their government’s incentive to protect all citizens through centralized actions. “This is not the way we want to go—to put the responsibility for breathing clean air on the individual instead of on the state,” she says. Instead, she argues, the state or central government can be much more effective at providing clean air and water to rich and poor residents alike through the passage and enforcement of adequate laws.

**Figure:**
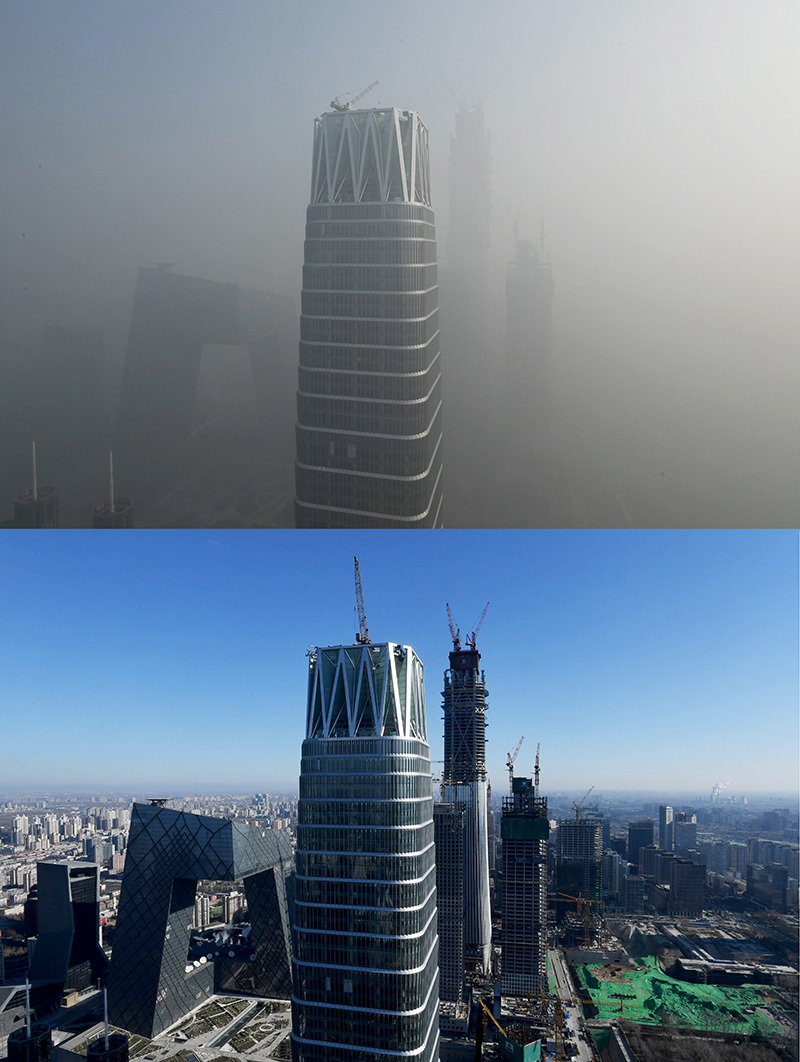
These photos show the air quality in Beijing on 1 January 2017 (top) in the midst of an “airpocalypse” smog emergency and on a clear day the week before. The notoriously bad air in many Chinese cities is slowly improving overall, but long-term exposures are still many times higher than World Health Organization recommendations. © Greg Baker/AFP/Getty Images

The ethical line between centralized and distributed (i.e., individualized) solutions is a fine one, suggests Nino Künzli, deputy director of the Swiss Tropical and Public Health Institute and dean of the Swiss School of Public Health. “We have to acknowledge that there are individual rights and governmental duties,” he says. “The success of the latter may take years to fully materialize. In the meantime, individuals have the right to know how to protect themselves.”

This right, however, does not guarantee awareness of the risk in the first place, the financial means to respond to it, or accurate knowledge of what protective measures to take.^4^ Even for those who can afford them, individual efforts like personal breathing masks and in-home water filters are no guarantee of safety, as they can still be ineffective if chosen or used improperly. Partially as a result, studies suggest, exposures to harmful chemicals through air and water around the world are distributed unevenly—and often inequitably.^5^

## Air Pollution Exposures

The notoriously bad air in many Chinese cities is slowly improving overall, but long-term exposures are still many times higher than World Health Organization recommendations.^6^ ^,^ ^7^ And seasonal spikes can be far worse. At least once every winter, it seems, a Chinese “airpocalypse” grabs international headlines as coal burning escalates and weather patterns trap polluted air, subjecting citizens to visibly heavy, hazardous smog.^1^

Media reports often include photos of individuals who have turned to breathing masks—flimsy, mostly useless surgical masks as well as tighter-fitting particulate respirators—to protect themselves from the dangerous air. For many Western viewers, these images may be a symbol of the country’s failure to keep air pollution in check during its recent industrialization. For others, the masks may be an accepted part of modern life in rapidly developing nations like China and India: a small price to pay for lifting millions out of poverty. After all, countries in the developed world also experienced extreme pollution as they industrialized during the eighteenth and nineteenth centuries.^8^

The better masks can have benefits, even for healthy individuals.^9^ In a recent study evaluating the short-term cardiovascular health effects of wearing particulate respirators, researchers from Fudan University in Shanghai and Texas A&M University’s School of Public Health measured heart rate variability and blood pressure in 24 healthy young adults with and without masks. Half the participants wore their respirators whenever possible for 48 hours, while the other half went mask-free. Three weeks later, the randomly assigned groups swapped places. All participants wore devices that measured their blood pressure throughout each 48-hour period.

Across both groups the researchers found respirator use to be associated with healthy blood pressure and increased autonomic nervous function, both indicators of good cardiovascular health. This was true at a time when concentrations of fine particulate matter averaged 74.2 µg/m^3^, which is three times the WHO daily standard (yet only a fraction of what Beijing saw at the start of 2017^10^).

Künzli welcomes the study’s findings even as he laments the need for respirators. “They are not the solution in China, but they give some reduction in exposure,” he says. “I don’t think we should fundamentally question that in the context of these highly polluted megacities where people have no other choice. … In that sense I have to say I appreciate these scientists critically evaluating wearing masks and what kind of mask we need to wear to get any benefit.”

**Figure:**
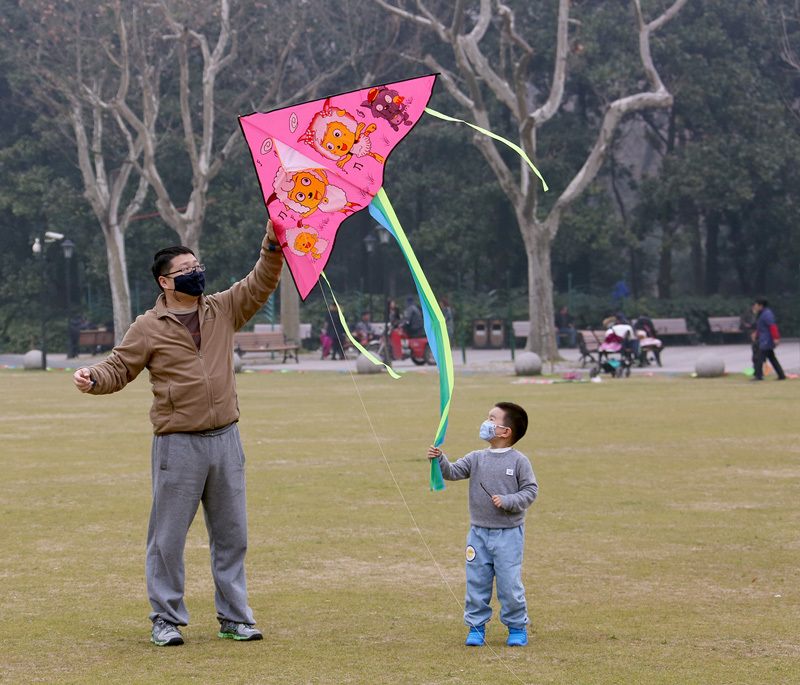
This father and son in Shanghai wore masks on a day in 2015 when the city’s Air Quality Index was 2.3 times the limit considered healthy. Chinese residents often turn to breathing masks to protect themselves from dangerous air. Tight-fitting particulate respirators can effectively block airborne pollutants, but the more commonly used surgical masks are mostly useless. © VCG/Contributor/Getty Images

Such information is sorely needed in China, believes Zhuohui Zhao, a coauthor of the paper and an associate professor in Fudan University’s School of Public Health. The mask tested in the study was 3M’s 8210V model, which is an N95 respirator, meaning it filters out at least 95% of solid and water-based particulates up to 0.3 µm in diameter.^11^ But the 8210V is just one of countless options available on the market. While breathing masks can vary widely in performance and fit, with cheap and ineffective surgical masks perhaps the most widely used, the differences among them are not always apparent to the consumer, Zhao says.

“For common people, it’s really hard to identify what kind of mask is useful or what kind of air purifier is efficient,” she says. “That’s true even for people working at universities. I have staff and friends in other colleges who say they are very confused about the market. There are many new brands of air purifiers, and there are lots of masks for them to choose from.”

Zhao says the 8210V retails in China for US$1.50–2.00 each. The nation’s average yearly salary is US$9,000,^12^ putting regular use of effective N95 respirators within reach of the middle and upper classes but potentially not the poor.

Many other people in China choose not to wear any kind of mask at all—a group that may be larger than some expect, Zhao believes. She says this is due in large part to a low perception of risk arising from limited knowledge of the potential adverse health effects of particulate matter. “People that know more about the pollution tend to take protective actions,” she explains. “But this group of people is still a small part of the population.”

Reliable statistics on mask usage are hard to come by, Zhao says, but observations from Beijing reported in January 2017 suggest regular mask wearers are indeed a minority.^13^ Over the course of five weeks in the preceding November and December—a time of year when local air quality is typically at its worst—daily usage rates along two streets in the capital ranged from about 10% to 50%. Nevertheless, other reports suggest risk perception is on the rise nationwide.^14^

While some companies in China’s polluted cities distribute respirators as an employee benefit, Zhao notes, no such subsidy exists for the unemployed or working poor. Nor would Hoffman, Künzli, or their colleagues in epidemiology and public health accept such a policy, they say. “If that was part of the official strategies of governments, of course we would need to step up very loudly and make clear that this was a wrong way of going forward,” says Künzli. “I want to see governments invest in cleaning up the air.”

## Water Pollution Exposures

Drinking water comes with its own set of expectations around centralized versus distributed approaches to risk mitigation. In the United States, the Safe Drinking Water Act requires that water delivered to ratepayers by public water systems meet high health-based standards for both chemical and microbial contaminants.^15^ Yet this doesn’t always guarantee the elimination of all harmful agents.

One growing concern among citizens and many local governments in recent months has been the safe delivery of drinking water through lead-bearing plumbing materials. Such materials were banned under the Safe Drinking Water Act Amendments of 1986 but remain in use in many older homes and buildings.^16^

Lead is just one of many potential threats facing the 150,000 public water systems in the United States. The federal Safe Drinking Water Act regulates 80 different chemicals including disinfection by-products, organic and inorganic chemicals, radionuclides, and six microorganism groups, including *Cryptosporidium* and coliforms.^17^ States may impose their own additional standards.

Most large water systems in the nation have few problems meeting these standards. That’s less true for small systems (often defined as those having fewer than a few thousand connections), which serve about 38 million people, or 12% of the U.S. population.^18^ “Noncompliance occurs more frequently at smaller public water systems because they often have fewer resources to operate and maintain compliance,” states a 2015 U.S. Environmental Protection Agency (EPA) report on the nation’s public water systems.^19^

**Figure:**
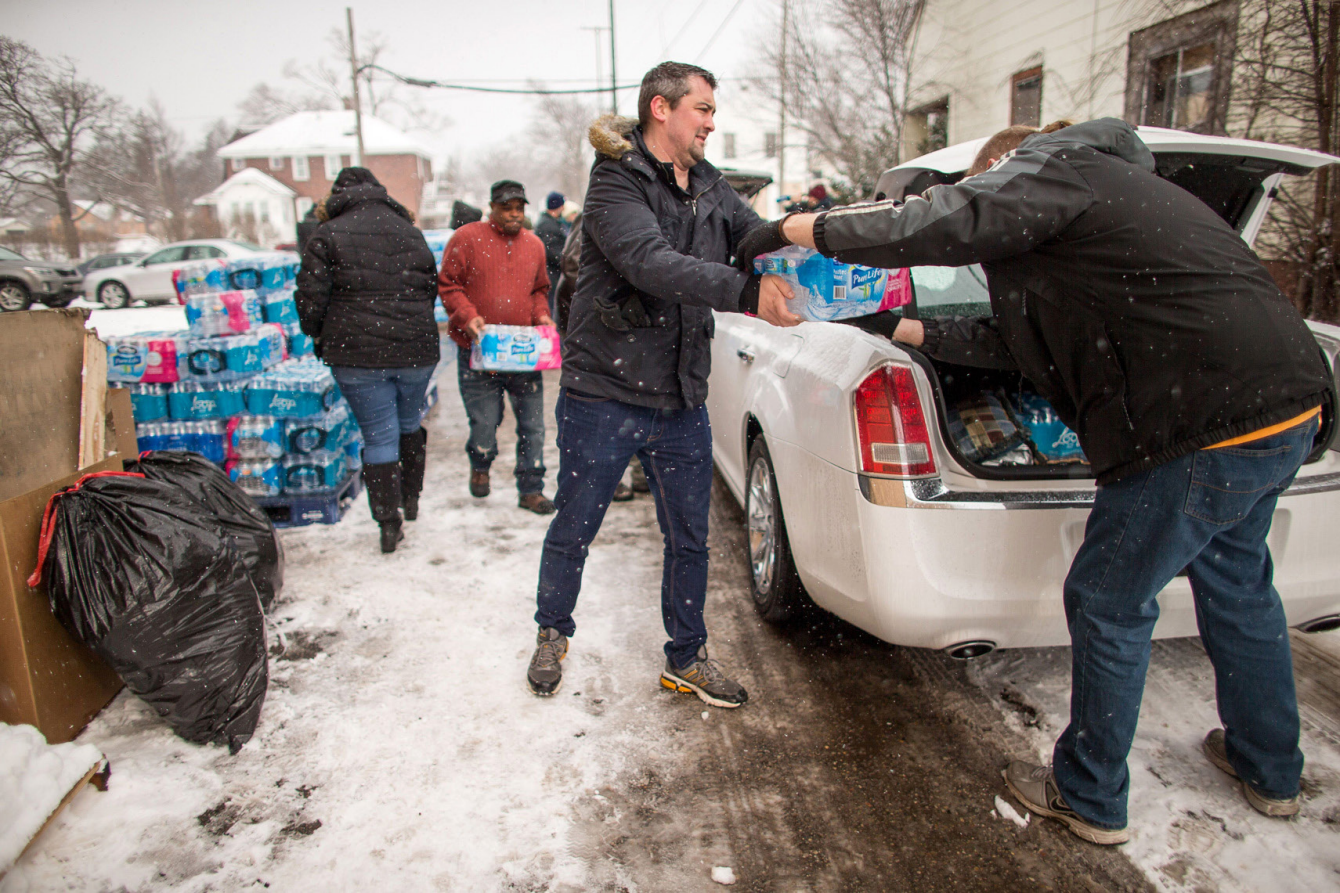
Volunteers loaded cases of free water into waiting vehicles in Flint, Michigan, as part of an effort to provide potable water to residents affected by lead contamination. Programs like Flint’s that use public funds to subsidize stopgap measures during an emergency appear to be on solid ground ethically—provided all residents have equal access. © Geoff Robins/AFP/Getty Images

Arsenic, for example, is a carcinogen that can be prohibitively expensive to remove centrally in small systems lacking economies of scale, says John Pujol, CEO of water-testing firm SimpleWater, Inc., which holds a license for a proprietary arsenic remediation technology it hopes to commercialize in the United States. As a result, arsenic often goes untreated in such systems, even at levels known to be unsafe and that significantly exceed the federal limit of 10 ppb.^19^

That’s what has happened in nearly 100 small systems serving 55,000 people in California alone, according to a recent report by the Environmental Integrity Project, an environmental watchdog group.^20^ Public water systems that fall out of compliance with EPA standards may receive warning letters or notices of violation, or in more severe cases be subject to citations, administrative orders, criminal charges, or other sanctions.^19^

Many Americans simply don’t trust centralized treatment systems of any size to deliver safe water to their homes, suggests Joseph Cotruvo, a public health consultant and former director of the EPA Drinking Water Standards Division. A 2015 national telephone survey by the Water Quality Association, which represents the water treatment industry, found that 59% of respondents were highly concerned about contaminants in their drinking water, 43% used a water filter, and 70% maintained that the municipality, not the individual, still bore ultimate responsibility for drinking water safety.^21^

“If you look at public perception and concerns, a very high percentage of people have negative perceptions of their drinking water,” says Cotruvo. “So they’re voting with their pocket books, buying bottled water or filters.” Yet Cotruvo, whose tenure at the EPA began in the agency’s earliest days, also believes that in some cases this perception is misdirected: “Municipal water in the U.S. is actually safer than ever, especially since the implementation of the Safe Drinking Water Act,” he says.

That said, the recent crisis in Flint, Michigan, did not help public perception of centralized water treatment. It also revealed the challenges of distributed treatment on a large scale. In late 2015, a full year and a half after switching its water source from the Detroit Water and Sewerage Department to the corrosive Flint River and setting off perhaps the highest-profile drinking water crisis this country has ever seen, the city began offering free filters to residents concerned about the safety of their water. This improved people’s access to safe water but only among those who were already aware of and able to act on the problem.^22^ ^,^ ^23^ ^,^ ^24^

Two months later, Flint mayor Karen Weaver expanded the program’s reach by declaring an official emergency and advising residents to drink only bottled water or filtered tap water.^25^ The state soon followed suit and began ramping up the distribution of bottled water, filters, replacement cartridges, and at-home test kits through official centers and limited home delivery.^26^ However, it was not yet absolved of the responsibility to deliver safe drinking water to all residents.

In November 2016 a federal court asserted as much by ordering the home delivery of bottled water to any Flint resident lacking a verified water filter. City and state officials twice fought the order—saying it would cost Michigan at least $10.45 million a month—but lost.^27^ ^,^ ^28^ ^,^ ^29^ On 24 January 2017, state officials reported that lead levels in Flint’s water were finally back below federal limits,^30^ but to date the delivery order stands—as does the city’s own recommendation to use a filter.^31^

Aside from lead, other agents and factors raise new questions about what constitutes drinking water safety. These include emerging chemicals of concern that cannot always be removed, such as pharmaceuticals^32^ and nanoparticles^33^; aging infrastructure under streets and inside homes; and other persistent threats, such as nitrates^34^ and *Legionella* bacteria.^35^

At the heart of these issues is a philosophical and practical question about the role of centralized versus distributed solutions within public water systems. The centralized water treatment plants and distribution systems built in American cities and suburbs over the last 100 years were designed under the premise that in-home treatment shouldn’t be necessary, says David Sedlak, a professor of environmental engineering at the University of California, Berkeley. That view persists, he says, even as weaknesses of the centralized model have emerged in recent years.

## Mitigation Challenges

If the most ethical solutions to air pollution are always centralized, with drinking water it’s not so black and white. Programs like Flint’s that use existing public funds to subsidize filters and bottled water during an emergency appear to be on solid ground ethically—provided, of course, all residents have equal access. A public water system that is out of compliance with federal water standards and delivering potentially unsafe water, by contrast, places the burden squarely on individuals to be aware of and then attempt to mitigate the problem themselves.

Still, most experts don’t recommend that large or even medium-size water systems seek to systematize point-of-use treatment—that is, gain EPA approval—because it can become a logistical (and financial) nightmare. In their appeal of the federal order to deliver bottled water to any of Flint’s 100,000 residents without an approved filter, state officials said such a plan would require a “Herculean effort” and increase the scope of Michigan’s emergency response “to an unnecessary and insurmountable degree.”^28^

**Figure:**
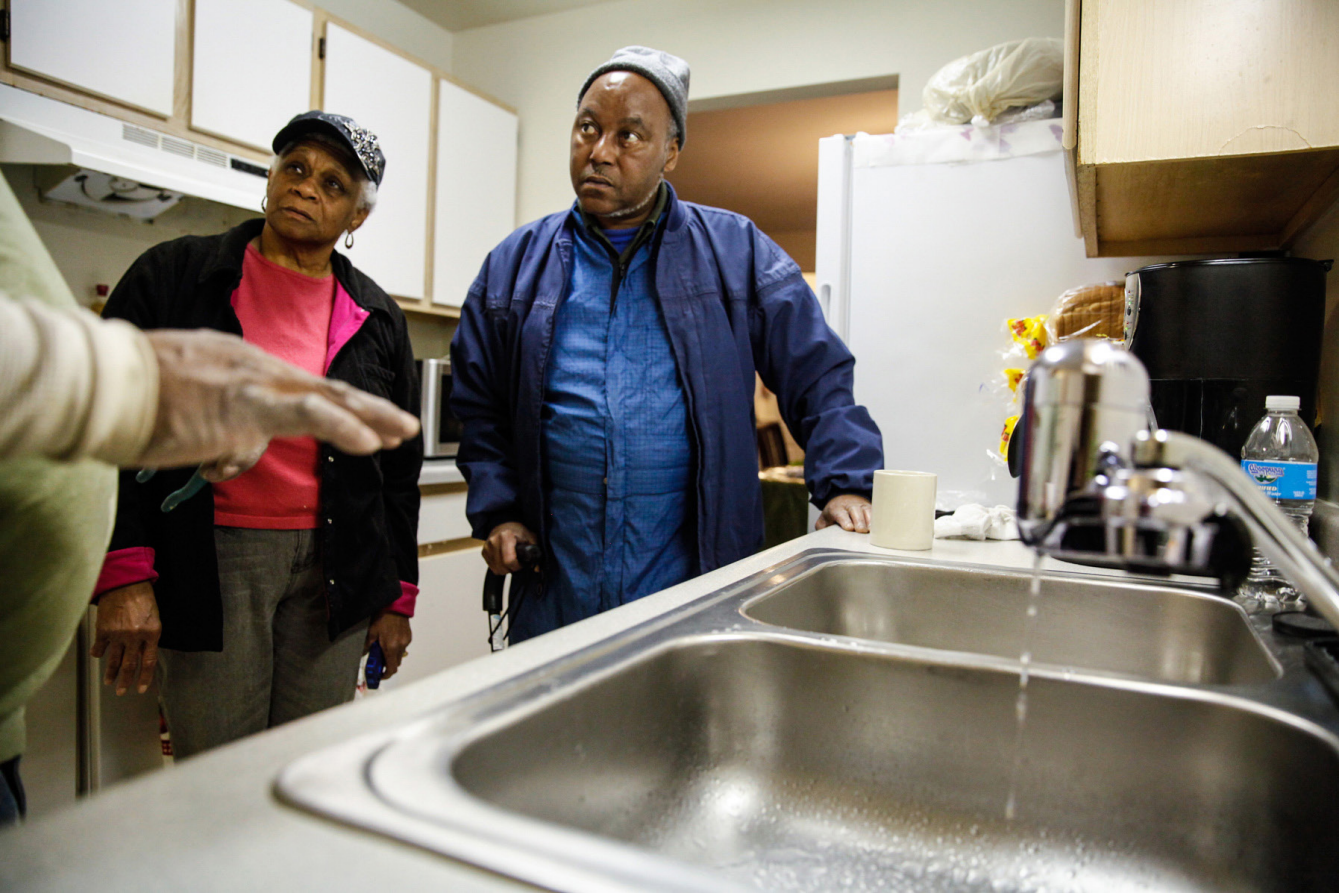
Residents in Flint learned about their new faucet-mounted filter from the handyman who installed it. The Flint crisis has illustrated the financial and logistical challenges of distributed treatment on a large scale. However, some experts believe point-of-use treatment strategies can be an affordable and efficient option for small water systems. © Sarah Rice/Getty Images

Instead, experts including Cotruvo believe reverse-osmosis units and other under-sink or faucet-mounted filters represent an attractive option for small, often cash-strapped systems across the country hoping to provide safe water and stay in compliance with federal and state regulations.^36^ “There is an economy of scale level where central treatment is more cost-effective, although still expensive in a small community compared to a large community,” he says. Point-of-use strategies are “a more efficient way of providing safe water because you’re really only treating the water that people consume.”

Yet a bevy of regulatory and economic hurdles can stand in the way of this strategy. According to federal policy, the water system operator or utility bears all responsibility for purchasing, installing, and maintaining the devices, including inspecting every unit annually.^37^ This entails routine visits inside private homes at considerable expense and effort that climbs rapidly with the number of connections.

“The problem is that it requires a pretty sophisticated maintenance and regulatory apparatus that doesn’t currently exist,” says Pujol. There is also a problem with perception, notes Sedlak. He explains, “There is this belief among people who are involved in centralized water systems that moving toward point-of-use is regressing and is basically admitting that we can’t deliver safe water in a centralized system.”

If point-of-use treatment were to be more readily accepted at the federal and state levels, Cotruvo believes, many people nationwide could quickly gain access to safer water. Instead, only a tiny number of water systems nationwide—fewer than 100 out of 150,000, Cotruvo estimates—have sought and gained approval to use distributed solutions to maintain safe water system-wide. Many others simply fall out of compliance with federal law.

The EPA reports that in 2013, the most recent year for which it has tabulated data, 27% of all public water systems—serving roughly a quarter of the U.S. population—had at least one “significant” violation of the Safe Drinking Water Act, a category that includes both technical and health-based violations.^18^ More than two-thirds of these were related to monitoring and reporting, which the EPA considers a serious violation because it makes it impossible to know whether drinking water standards are being met.

Since out-of-compliance systems are more likely to be small, and small systems are more likely to serve rural and low-income communities, the current system puts citizens who are already underserved at a potential disadvantage. Additionally, contaminants such as naturally occurring arsenic and agricultural chemicals are more likely to be present in lower-income regions like the rural Midwest and California’s Central Valley.^38^ “Many members of the low-income and environmental justice [advocacy] community take point-of-use seriously, because they see it as pretty much the only way forward,” Pujol says.

Peter Gleick, president emeritus and chief scientist of the nonprofit Pacific Institute, says he believes recent developments have led U.S. public water systems to a fork in the road. Down one path is the potential to rebuild water systems to a higher standard, including through less-centralized approaches.

Down the other is a future he describes as a “downward spiral in the quality and cost of our water systems,” where the rich install point-of-use systems and the poor are left relying on bottled water or drinking whatever comes out of the tap. Already, Gleick says, poor infrastructure, rural water contamination, and poverty threaten basic water services for millions of Americans. “Inequitable access to safe and affordable drinking water is a large and growing problem,” he says, “and should be an embarrassment for a country like the United States.”
